# Sugar and Hormone Dynamics and the Expression Profiles of SUT/SUC and SWEET Sugar Transporters during Flower Development in *Petunia axillaris*

**DOI:** 10.3390/plants9121770

**Published:** 2020-12-14

**Authors:** Junaid Iftikhar, Meiling Lyu, Zhuoyi Liu, Nasir Mehmood, Nigarish Munir, Mohamed A. A. Ahmed, Wajjiha Batool, Mehtab Muhammad Aslam, Yuan Yuan, Binghua Wu

**Affiliations:** 1 College of Horticulture and the Fujian Provincial Key Laboratory of Plant Functional Biology, Fujian Agriculture and Forestry University, Fuzhou 350002, China; 2161971001@fafu.edu.cn (J.I.); mllyu@fafu.edu.cn (M.L.); Zhuoyi_Liu@fafu.edu.cn (Z.L.); 2151905001@fafu.edu.cn (N.M.); 2161906001@fafu.edu.cn (M.A.A.A.); yuanyuan@fafu.edu.cn (Y.Y.); 2 Institute of Horticultural Biotechnology, Fujian Agriculture and Forestry University, Fuzhou 350002, China; 2181905002@fafu.edu.cn; 3 College of Plant Protection, Fujian Agriculture and Forestry University, Fuzhou 350002, China; 2171916003@fafu.edu.cn; 4 College of life Sciences, Joint International Research Laboratory of Water and Nutrition in Crops, Fujian Agriculture and Forestry University, Fuzhou 350002, China; 2171916002@fafu.edu.cn

**Keywords:** *Petunia axillaris*, flower development, sugar, phytohormones, sugar transporter, gene expression

## Abstract

Flowering is the first committed step of plant sexual reproduction. While the developing flower is a strong sink requiring large quantity of sugars from photosynthetic source tissues, this process is under-temper-spatially controlled via hormone signaling pathway and nutrient availability. Sugar transporters SUT/SUC and SWEET mediate sugars movement across membranes and play a significant role in various physiological processes, including reproductive organ development. In *Petunia axillaris*, a model ornamental plant, 5 *SUT*/*SUC* and 36 *SWEET* genes are identified in the current version of the genome. Analysis of their gene structure and chromosomal locations reveal that *SWEET* family is moderately expanded. Most of the transporter genes are abundantly expressed in the flower than in other organs. During the five flower developmental stages, transcript levels of *PaSUT1*, *PaSUT3*, *PaSWEET13c*, *PaSWEET9a*, *PaSWEET1d*, *PaSWEET5a* and *PaSWEET14a* increase with the maturation of the flower and reach their maximum in the fully open flowers. *PaSWEET9c*, the nectar-specific *PhNEC1* orthologous, is expressed in matured and fully opened flowers. Moreover, determination of sugar concentrations and phytohormone dynamics in flowers at the five developmental stages shows that glucose is the predominant form of sugar in young flowers at the early stage but depletes at the later stage, whereas sucrose accumulates only in maturated flowers prior to the corolla opening. On the other hand, GA_3_ content and to a less extent IAA and zeatin decreases with the flower development; however, JA, SA and ABA display a remarkable peak at mid- or later flower developmental stage.

## 1. Introduction

Being the principal product of photosynthesis, sugars in the form of sucrose are translocated from source organs (leaves) to sink organs (modified leaves, roots, seeds, and reproductive organs) via phloem sap [1,2,3]. The availability of sugars for various mechanisms in plants has profound impacts on plant growth and development [4,5,6]. Evidence emerging in recent years implies that sugars can also act as signaling molecules that control distinct aspects of plant development and the cross-talk between sugar-based signaling pathways and various phytohormones play important roles in modulating plant growth and development [7]. Flowering is a major reproductive-development event in higher plants that under strictly controlled by endogenous signaling pathways influenced by sugars availability and phytohormones interactions [8]. After the floral initiation or vegetative-to-reproductive transition, the floral bud undergoes differentiation and ontogeny to build up a flower or an inflorescence and becomes an active sink that requires large net influx of sugars from the photosynthetic leaves.

Efflux and influx of sugars across the cellular and subcellular compartments are facilitated by sugar transport proteins. Plant genome encodes at least three families of sugar transporters: monosaccharide/polyol transporters (MSTs), sucrose transporters (SUTs or SUCs), and SWEETs [9] [10]. MSTs are associated with sugar influx and transport glucose and other substrates [11], while SUTs and SWEETs play a significant role in phloem loading and unloading [2]. However, SWEETs are bidirectional sugar transporters and mainly involved in sugar efflux [12,13].

The SUTs in plants are related to the glycoside-pentoside-hexuronide (GPH) cation symporter family that belongs to the major facilitator superfamily (MFS) and are typically encoded by three to nine *SUT/SUC* genes, e.g., in *Arabidopsis* 7 *SUC*-like genes and two pseudogenes, which are clustered into three types [3,14]. While type I SUTs, the eudicot-specific transporters, are mainly found in the plasma membrane of sieve element and companion cells to facilitate phloem loading, at least one of the members *AtSUC1* is required for pollen germination [15,16,17,18]. Type II SUTs are phylogenetically separated between dicot (IIA) and monocot (IIB) proteins and have demonstrated roles in phloem loading or unloading in both sink and source tissues [9]. Although most SUTs studied are plasma membrane-localized, some transporters from the type III SUTs are tonoplast proteins, for example the PtaSUT4 in Populus transport sucrose to vacuole for storage to control the cytosolic sucrose concentration [19,20]. Plasma membrane-localized type III SUTs proteins have also been implicated in sucrose signaling, in addition to the type II protein LeSUT2 and AtSUT2 [21,22,23].

The SWEETs transporter family is the newest identified class of sugar transporters previously recognized as *Medicago truncatula* nodulin 3 (MtN3)/saliva [24]. SWEETs belong to a superfamily of transporters containing seven transmembrane helices and they show a pair of structural repeats that folds into two transmembrane domains connected by a loop [13,25,26]. SWEETs are localized in cellular and organelle membranes [4,27,28], and are widely expressed in plant tissues [12,29]. Recent developments in whole-genome sequencing have resulted in genome-wide identification of SWEETs in an increasing number of plant species including cultivated crops, and fruits and vegetables of immense agricultural importance, such as rice (*Oryza sativa*) [30], wheat (*Triticum aestivum*) [31], soybean (*Glycine max*) [32], sorghum (*Sorghum bicolor*) [33], oilseed rape (*Brassica napus*) [1], grape (*Vitus vinifera*) [34], apple (*Malus domestica*) [35], banana (*Musa acuminate*) [36], cabbage (*Brassica oleracea*) [37], tomato (*Solanum lycopersicum*) [38] and potato (*Solanum tuberosum*) [39]. Typically, SWEET gene families in angiosperms contain 15 to 25 members, for example, 17 and 21 in arabidopsis and rice, respectively [40]. However, evolution in various plant species resulted in duplication of *SWEET* genes such as 30 in cabbage, 52 in soybean, 68 in oilseed rape and 121 in wheat. Functional characterization and biochemical analysis of SWEET sugar transporters have revealed their important roles in diverse physiological processes in plants, such as leaf senescence [41], plant nectar secretion [42,43], response to biotic, abiotic and hormonal stress [34,44,45,46,47,48], seed germination [49], and pollen, seed (filling) and fruit development [28,50,51,52,53,54]. The expression of *SWEETs* in the contest of regulation of source-to-sink sucrose transport is also modulated under different physiological conditions [55].

Once imported into the developing flower, sucrose is cleaved to glucose and fructose or otherwise storage intracellularly. The conversion and utilization of sugar molecules is dynamically controlled by the feedback of metabolism and morphogenesis processes [56]. The development from a tiny bud to a fully expanded flora organ, the mature flower, involves not only the cell division and proliferation but also the gain of specialized traits like color, scent and taste which are vital for the success in pollination and fertilization [57]. Given the complexity in the development of the flower, the regulatory network of sugar metabolism in this process is far from fully explored. While extensive functional studies of transporters in floral tissues is carried out in the model plants *Arabidopsis* and rice, fewer investigation in other species with agricultural or horticultural importance are reported so far.

*Petunia* is an important ornamental plant world-wide and is emerging also as an ideal model for genetic study pertaining to ornamental traits [58]. Here we report the whole-genome identification of *SUT* and *SWEET* genes and their tissue-specific expression in the wild-type *Petunia axillaris*. We also analyze the flower development-associated transcript abundance of the transporters with respect to the soluble sugar content and the main plant hormone accumulation.

## 2. Results

### 2.1. The Five-Gene Family of SUT/SUC Transporters in P. axillaris

The currently available genomic sequences in *P. axillaris* provide a base for searching the complete set of genes encoding sugar transporters. Through homologous search and manual curation, 5 *SUT* and 36 *SWEET* genes were identified in the *P. axillaris* genome version 1.62 [59]. These genes were then designated as *PaSUT1* to *PaSUT5* and *PaSWEET1a* to *PaSWEET 17* based on *Arabidopsis* orthologous sequences, respectively (Table 1). Using three different bioinformatic prediction programs, the protein subcellular localization was also analyzed, which showed that the five SUTs were most likely localized to plasma membrane while considerable numbers of the SWEETs seemed to be endomembrane system-associated transporters, especially in the vacuolar membrane (tonoplast) (Table S1).

**Figure 1:**
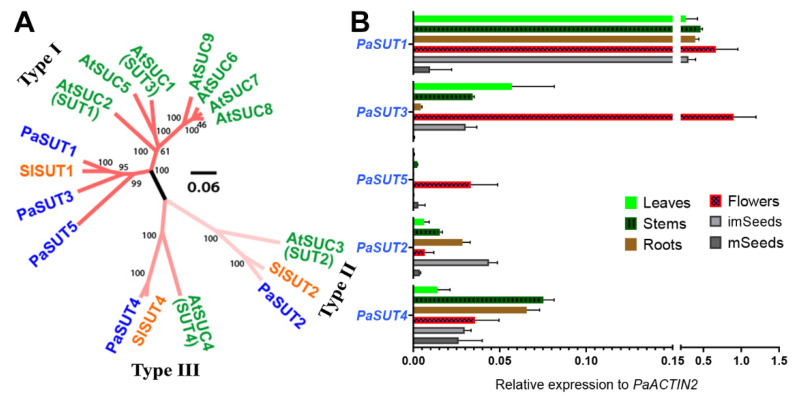
Phylogenetic analysis and tissue-specific expression profile of the *P. axillaris* SUT/SUC sugar transporters. (**A**) The 5 SUT/SUC sequences fall into three groups, representing the type I, Type II and Type III members. PaSUTs are shown in blue. *Arabidopsis thaliana* orthologs are in green: AtSUC1, At1g71880; AtSUC2, At1g22710; AtSUC3, At2g02860; AtSUC4, At1g09960; AtSUC5, AT1G71890; AtSUC6, AT5G43610; AtSUC7, AT1G66570; AtSUC8, AT2G14670; AtSUC9, At5g06170. *Solanum lycopersicum* orthologs are in orange: SlSUT1, NP_001289830.1; SlSUT2, NP_001234321.2; SlSUT4, NP_001234344.2. Sequence alignment and analysis are conducted using MUSCLE with default parameters, and the phylogenetic tree is constructed with MEGA X version 10.1.8 using the neighbor-joining (NJ) method with 1000 bootstrap replications. The tree is graphed via Figtree version 1.4.4, with bootstrap value greater than 50% shown. The scale bar represents the ammino acid substitution rate per site. (**B**) Tissue-specific gene expression analysis of the SUT/SUC genes via quantitative real-time PCR. Data are mean of 3 biological replications with the error bars representing SD.

The small *SUT/SUC* gene family in *P. axillaris* consists of five members. Similar to the situation in *Arabidopsis*, both Type II and Type III *SUT* have only one single member, *PaSUT2* and *PaSUT4*, respectively. The other three members, *PaSUT1/3/5*, belong to the Type I subfamily which has seven counterparts in *Arabidopsis* (Figure 1A). These 5 *PaSUT* genes are located at separated scaffolds of the current genome version (Figure S1A). The intron-exon structure of the *SUT* genes is somehow diverse, with the longest and shortest sequences being found for *PaSUT4* (11907 bp) and *PaSUT1* (4166 bp), respectively (Figure S2A).

With the whole-genome identification of the *PaSUT* genes, we were able to examine the mRNA abundance in different organs and tissues, including leaves, stems, roots, flowers (fully opened), immature seed and mature seeds.

Among the five members, *PaSUT1* is the most highly expressed gene in almost all tissues tested, except in mature seeds, which contains very low detectable mRNA for all *PaSUTs*. Similar to *PaSUT1*, the other two Type I genes *PaSUT3* and *PaSUT5* also display a preferential expression in flowers. Yet quantitatively, expression of the *PaSUT5* is much lower as compared to both *PaSUT1* or *PaSUT3*. The *PaSUT5* seems to be rarely expressed in most tissues except for flowers. On the other hand, *PaSUT2* is preferentially expressed in immature seeds, roots and stems, whilst *PaSUT4* is similarly expressed in most tissues (Figure 1B).

### 2.2. The Genome of P. axillaris Encode 36 Genes for the SWEET Transporters with Disparate Tissue Expression Profile

**Figure 2:**
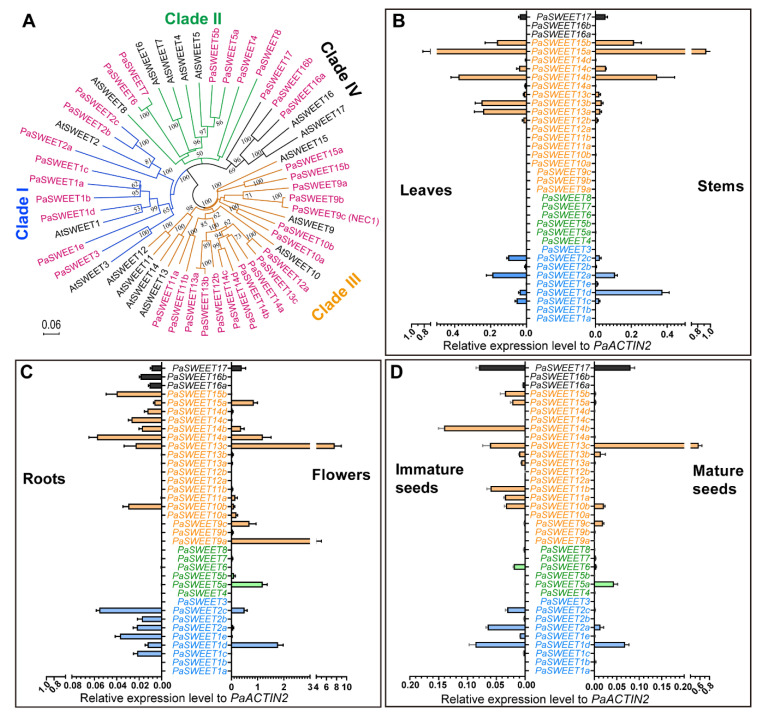
Phylogenetic analysis and tissue-specific expression profile of the *P. axillaris* SWEET transporters. (**A**) The 36-gene family of SWEET proteins are grouped into four clades (depicted in colored branch lines) together with the well-annotated *Arabidopsis* transporters. Sequence alignment and analysis are conducted using MUSCLE with default parameters, and the neighbor-joining (NJ) tree is constructed using MEGA X with 1000 bootstrap replications and visualized via Figtree version 1.4.4, with bootstrap value greater than 50% shown. The scale bar represents the ammino acid substitution rate per site. (**B**–**D**) Tissue-specific gene expression analysis of the SWEET genes via quantitative real-time PCR. Data are mean of 3 biological replications with the error bars representing SD.

The *SWEET* family in *P. axillaris* is considerably expanded which includes 36 members, given that angiosperm species with known genome sequence normally contain between 15 and 30 *SWEET* genes [40]. The *Petunia* genes are well separated in the four clades each containing 9, 6, 18 and 3 genes, respectively, as supported by the phylogenetic relationships. For a comparison, clade-specific members in the 17-gene-family in *A. thaliana* are 3, 4, 7 and 2, respectively **(**Figure 2A). Analysis of the chromosomal location of the *PaSWEET* genes indicates that at least three tandem-gene-locus clusters exist for *SWEETs*. One three-gene cluster is found for *PaSWEET1e/Peaxi162Scf00263g01423*, *PaSWEET1d/Peaxi162Scf00263g00156* and *PaSWEET1a/ Peaxi162Scf00263g00161* within a ~20 kb region of the same scaffold, oriented in the same direction (Figure S1B). A second tandem gene array contains six *SWEET* sequences (*PaSWEET12a*, *PaSWEET14c*, *PaSWEET13c*, *PaSWEET14d*, *PaSWEET14a* and *PaSWEET11a*) located in a ~113kb region of the scaffold00516. Other tandem genes are *PaSWEET16b* and *PaSWEET17* that together occupy a ~12 kb region (Figure S1B). It is likely that tandem gene duplication or triplication events may contribute to the expansion of *SWEET* family, especially in the clade I and III, in *P. axillaris* [59]. In the current version of the genome annotation, *PaSWEET4/Peaxi162Scf00102g01855.1* and *PaSWEET11a*/*Peaxi162Scf00516g00125* are incorrectly predicted resulting in short mRNAs. We made a new prediction by including the neighboring region to obtain the true full-length mRNA and protein sequences via web-based gene structure prediction tool FGENESH [60], yielding the corrected full-length *PaSWEET* sequences (Table 1).

Within each clade, the intron-exon structure of the *PaSWEET* genes seems to be similar among close orthologs. For example, genes *PaSWEET2a, PaSWEET2b, and PaSWEET2c* all have six exons. Again, such similarity in intron/exon structure may hints gene replication events during evolution, e.g., gene duplication or triplication. However, some closely related genes also display a significant difference in structural arrangements (Figure S2B). For instance, *PaSWEET14b* have only five exons, while its closely related *PaSWEET14a, PaSWEET14c,* and *PaSWEET14d* contain more than 15 exons. In addition, two *PaSWEET* genes have only one exon, namely *PaSWEET6* and *PaSWEET7*. Finally, most of *PaSWEETs* contain five to six exons, except the six genes *PaSWEET5a, PaSWEET12a, PASWEET12b, PaSWEET13c, PaSWEET14a, PaSWEET14c and PaSWEET14d*, which contained 16, 23, 22, 19, 31, 20 and 17 exons, respectively (Figure S2B).

The organ-specific expression profiles of the 36 *PaSWEET* genes reveal that the flower organ remains a major site for active transcription of these bidirectional sugar transporters, in which the gene expression levels are relatively higher as compared with that in the other tissues (Figure 2B–D). Specifically, *PaSWEET13c*, *PaSWEET9a*, *PaSWEET14a*, *PaSWEET15a* and *PaSWEET9c* are among the highly expressed clade III genes, while *PaSWEET1d*, *PaSWEET5a* and *PaSWEET17* are the most abundantly expressed genes from clade I, II and IV, respectively (Figure 2C). In the developing or mature seeds, mRNA of single or multiple members from each clade could be detected. During the maturation of the seeds, expression levels of clade I and clade IV members (*PaSWEET1e, 2a, 2c and 17*), as well as the *PaSWEET6* from clade II and *PaSWEET11a*, *11b*, *14b*, *15a* and *15b* from clade III decrease considerably, while those of *PaSWEET5a*, *9c* and *13c* are significantly upregulated (Figure 2D). It is also noted that transcripts of the clade II genes *PaSWEET5a* and *PaSWEET6* are only detected in reproductive tissues such as flowers and seeds (Figure 2C,D)

In the vegetative organs, transcripts of the clade II *SWEET* genes are hardly detected (Figure 2B,C). In leaves and stems, the expression profiles of *SWEET* genes are quite similar except those of *PaSWEET1d*, *PaSWEET13a* and *PaSWEET13b*, the former is expressed by ~ 7-fold more abundantly in stems while the latter two display higher mRNA levels in leaves than in stems by a factor of ~ 8-fold. Among all tissue tested, the root seems to express the lowest levels of *SWEET* genes (Figure 2B). However, the two clade IV members *PaSWEET16a* and *PaSWEET16b* are preferentially expressed in the root tissues and unmeasurable in all other tissues tested. Moreover, root tissues seem to express more single *PaSWEET* genes but the overall level of the transcripts is the lowest comparing to the other tissues (Figure 2C).

### 2.3. Expression Profiles of PaSUTs and PaSWEETs during Flower Development

**Figure 3:**
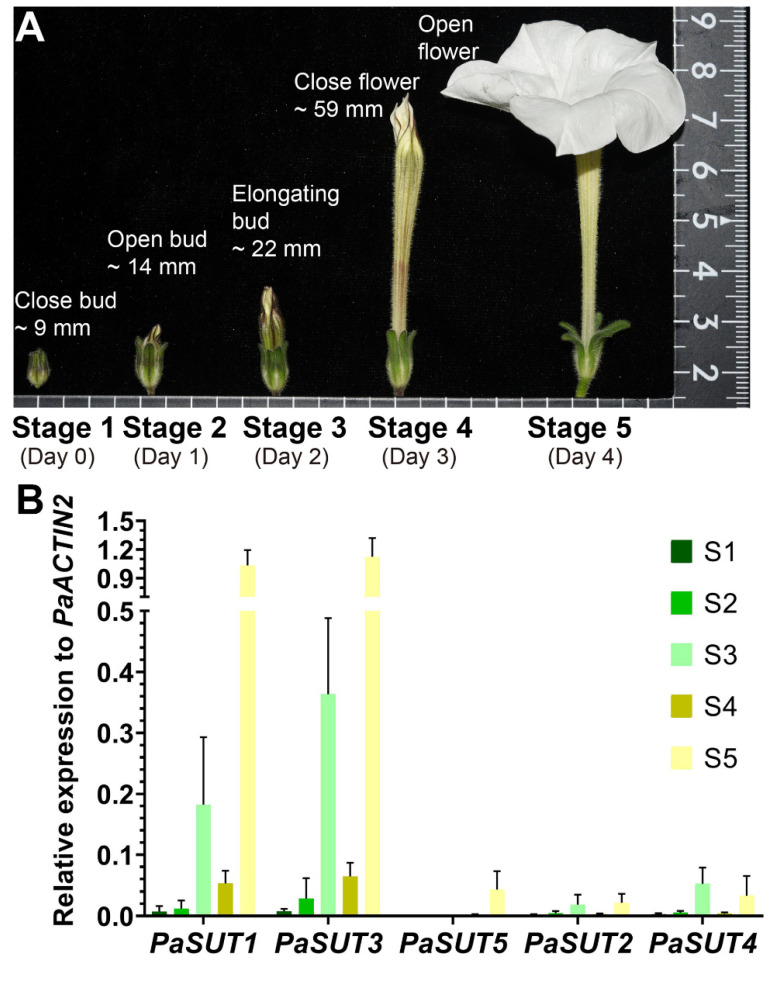
The *P. axillaris* flower developmental stages (**A**) and expression profile of the five *PaSUT* genes (**B**). Data represent mean and SD of three independent determinations, each is a pooled sample with 2-5 flowers.

The tissue-expression profiles of the two sugar transporter families indicated that the *Petunia* flowers are among the organs with highly transcribed sugar transporters. To refine the developmental effect on transporter gene expression, we further analyzed the stage-specific transcript accumulations. We divided the *Petunia* flower development into 5 stages after visible bud emergence (Figure 3A). Stage 1 of development is a green and close bud with a typical heigh of >9 mm. Stage 2 to 3 represent the elongating growth the *Petunia* corolla and stage 4 to 5 are characteristic of flower maturation and physiological shifts to pollinator attraction and fertilization. These stages are corresponded to the developmental stage 1 through 7 described in *Petunia hybrida* [61,62].

The five *PaSUTs* display a somehow similar expression pattern during the flower developmental stages, achieving maximal transcript level at corolla opening followed by a second higher peak of expression when the corolla starts to elongate at stage 3 (Figure 3B). However, *PaSUT5* is expressed only at stage 5, and together with *PaSUT2* and *PaSUT4*, these three have relatively lower transcript abundance in all stages of flower development. The remarkable upregulation of *PaSUT1* and *PaSUT3* during the initiation of corolla elongation and opening is a possible hint of large demands on sucrose import in these periods (Figure 3B).

**Figure 4:**
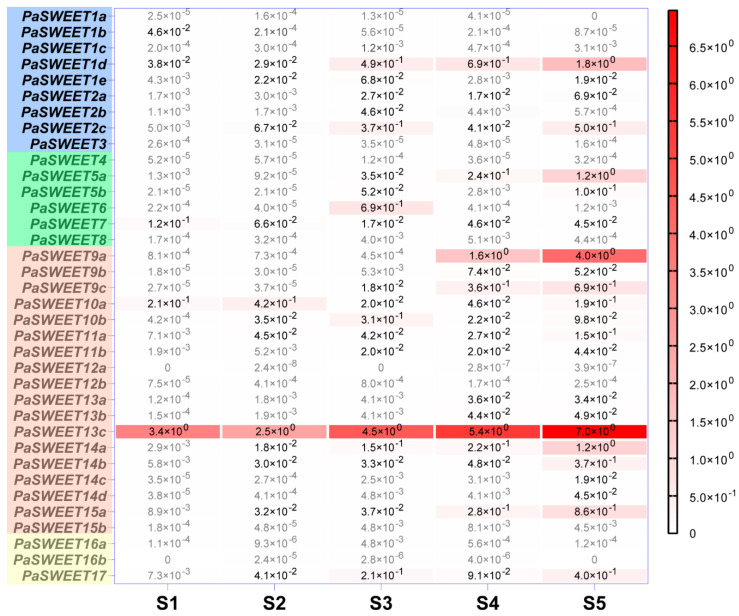
Quantification of 36 PaSWEET gene expression during flower development. Transcript abundance was determined via qRT-PCR using *ACTIN* as an endogenous reference gene. Data represent the average of three independent determinations. Each determination was run on pooled samples with 2–5 flowers. Values lower than 1.0 × 10^-3^ are depicted in grey for better visualization. The statistical parameters are provided in Table S2.

Two of the Clade IV *SWEET* genes, namely *PaSWEET16a* and *16b*, are hardly expressed in flowers of all stages, while only *PaSWEET17* shows detectable yet low level of transcript mainly at later stage of development. Among the six genes in the Clade II, *PaSWEET5a* is the highest expressed gene which is upregulated in the later stages, being 5- and 6.8-fold increases in stage 5 than in stage 4 and 3, respectively (Figure 4). Interestingly, *PaSWEET6* reaches its maximum expression at stage 3 significantly, whereas *PaSWEET7* shows a small expression peak in close buds at stage 1. Upregulation of transcription is also evident in Clade 1 *SWEET* genes, at least for *PaSWEET1d* and *PaSWEET2c*-both of which display highest transcript level at stage 5 (Figure 4).

The Clade III genes of *SWEET* family are probably among the most actively transcribed during *Petunia* flower development. *PaSWEET13c* and *PaSWEET9a* represent the two most highly expressed members of the whole family and are upregulated during development, reaching their maximum levels at the corolla opening stage 5 (Figure 4). The other Clade III members also show more or less upregulation during flower opening, except for *PaSWEET10a* and *PaSWEET10b* which exhibit higher expression at the early stages (Figure 4).

Taken together, these results indicate that genes of the sugar transporter families are actively transcribed during flower development in *petunia*, notably at the fully maturation stage ready for pollination and fertilization, but the abundance of mRNA levels does not necessarily correspond to the protein functions.

### 2.4. Phytohormone Dynamics and Sugar Contents during Flower Development

**Figure 5:**
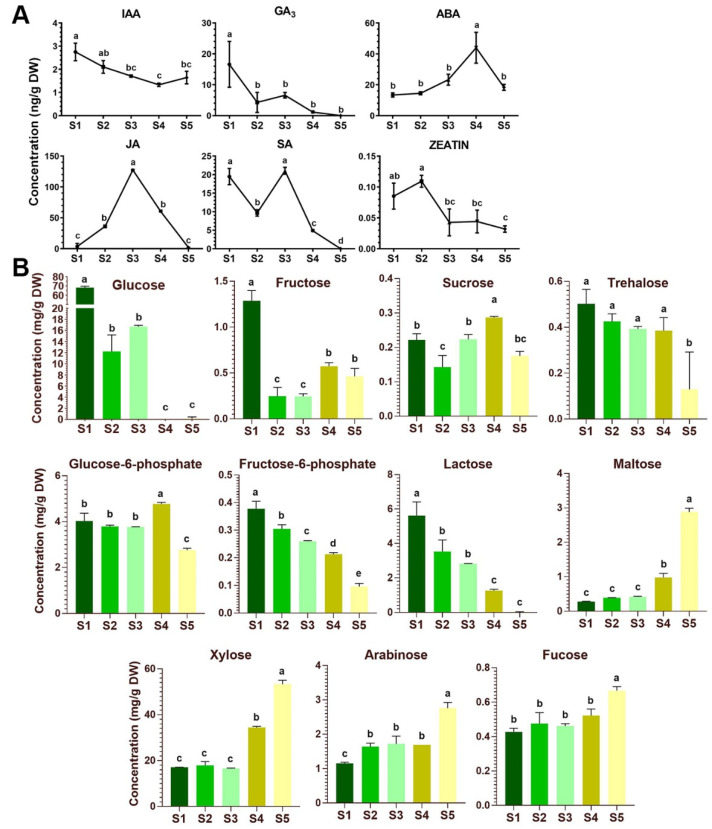
Phytohormone and sugar contents in the 5 stages during *P. axillaris* flower development. (**A**) Phytohormone dynamics during the flower development. Data are mean and SD of three independent experiments. (**B**) Sugar contents in the developmental stages. Date represent mean and SD of three independent experiments. Significant difference between group with variable letters above the line or bar are verified using two-way ANOVA and multiple *T*-test (*p* < 0.05) implemented in the Prism software version 7.04.

In stage 1 through stage 5 of the flower development, JA, ABA, SA, GA_3_, IAA and Zeatin were detected by a liquid chromatography-tandem mass spectrometry (LC/MS) analysis. Both IAA and GA_3_ show a declining trend, the concentrations of which in the green close buds are 2.75 and 16.60 ng g^−1^ DW but decrease to 1.64 and 0 ng g^−1^ DW in the open flowers at the stage 5, respectively (Figure 5A). In contrast, ABA content increase from stage 1 to stage 4 by ~2.6 folds and then drops significantly after the corolla is open. JA and SA are found to accumulate dramatically at stage 3 when the corolla just start to elongate and expend, although the SA concentration has another similar peak in the close bud of stage 1. Zeatin is detected in the *Petunia* flowers at relatively lower concentrations throughout the development, with a decline tendance after corolla elongation at stage 3. The maximal concentrations of JA, ABA and SA in the *Petunia* flowers are 127.41, 43.98 and 21.04 ng g^−1^ DW, being the three most abundant hormones (Figure 5A). These results highlight a dynamic feature of hormonal control in flower development.

To monitor the sugar content variation during *Petunia* flower development, a gas chromatography–quadruple time-of-flight mass spectrometry (GC-qToF-MS) approach was employed to analyze the nontargeted metabolome in the floral tissues. Soluble sugars (sucrose, fructose, glucose, trehalose and maltose), phosphorylated sugar intermediates (glucose-6-phosphate and fructose-6-phosphate) and other aldose sugars (xylose, arabinose and fucose) were identified in the extracts from flower tissues at the five stages of development (Figure 5B).

During flower development from the close buds to the fully open flowers, accumulations of four sugars, namely xylose, maltose, arabinose and fucose, gradually increase to their maximum of 53.26, 2.88, 2.76 and 0.67 mg g^-1^ DW in the fully open flower of stage 5, respectively, while the contents of glucose, lactose, fructose-6-phosphate and to a less extent of trehalose decline from stage 1 through stage 5, and the maximal concentration of these four sugars in the close bud are 68.78, 5.62, 0.38 and 0.50 mg g^−1^ DW, respectively (Figure 5B). However, the concentrations of sucrose, fructose, and glucose-6-phosphate in the flower tissues fluctuate at different stages (Figure 5B). Sucrose, the major long-distant-translocated sugar, is found to accumulate slightly at stage 4 when the flower reaches its maturation, whereas glucose and fructose, the two important hexoses delivered from sucrose cleavage, are significantly accumulated in close bud at stage 1 (Figure 5B). Interestingly, glucose seems to be the predominant forms of soluble sugar during early stages (stage 1 to 3) of flower development with the concentrations ranging from 12.22 to 68.78 mg g^−1^ DW, although its content declines rapidly to almost undetectable level at the later stages after flower maturation (stage 4 and 5). Moreover, being an important sugar intermediate, the concentrations of glucose-6-phosphate are relatively stable during early stages before flower maturation (stage 1 to 3) (Figure 5B).

## 3. Discussion

Unlike in *Arabidopsis* [63,64,65], the cellular events of flower development in *Petunia* are less documented. During the development of the *Petunia* flower, stage 1 to 2 is morphologically characteristic of a green bud of slowly growth rate with either close or open calyx, and these young flowers are undergoing floral organ differentiation, especially the development of stamens and carpels (Figure S3). We found that the contents of GA and SA are negatively, while that of JA is positively correlated to these two stages. The soluble sugar, particularly in the form of glucose, is greatly accumulated at the stage 1 but reduced at the stage 2. In stage 3-4, the corolla tube starts to elongate along with increase in length of stamen filaments. At stage 4, stamen, pistil and corolla reach the maximum in size and the flower become maturated with a long corolla tube. The stage 5 is characterized as fully opened flower and recognized with volatile emission and pollinator attraction. In these mid-age flowers, GA level is continually declined, which seem not correlated with a role in stimulating organ elongation as reported in *Arabidopsis* [66]. In a recent study in *P. hybrida* line P720, it is demonstrated that GA is negatively associated with scent release by repressing transcript levels of genes involved in floral phenylpropanoid scent production, and its level decline with the flower maturation and opening [66,67]. In addition to GA, our data also indicate that JA and SA peak significantly at stage 3 but decline sharply thereafter, which coincides with the glucose depletion at the latter two stages (Figure 3B,C). Moreover, ABA is remarkedly high at the maturation stage 4. Given that various hormones are known to play diverse roles during flower development [68,69,70,71,72], it will be necessary to further determine the potential cross-talk among the hormone signaling pathways in the regulation of the flower development.

The later stages of flower development display a depletion in glucose level but an increased accumulation of the cell-wall related monosaccharides, xylose, fucose and arabinose, as well as the starch-breakdown product maltose (Figure 5B), a disaccharide known to be metabolized to form glucose and hexose phosphates [64,65,73]. These alterations in sugar concentration are possibly linked to pollen formation, biosynthesis of nectar and scent during flower maturation and corolla opening. However, the molecular regulation is still not known. Although genes that function in *Petunia* flower development have been reported, including several MADS-box type floral meristem/organ identity genes [74] and a sympetaly-regulating gene *PhGATA19* [75], sugar transporter genes controlling the sugar dynamics in the flower are less studied. The hexose transporter *PhSTP1* was earlier characterized in the growing *Petunia* pollen tubes [76] and the *PhNEC1* (the *SWEET9c* in this study) was found to associate with nectar secretion phenotype, analog to the *Arabidopsis AtSWEET9* [42,77]. Recently, a transcriptome analysis of *P. axillaris* flowers identified two *SWEET* homologs, termed as *PaSWEET11* and *PaSWEET15* by the authors, which were expressed differentially during the flower development [78]. However, their sequence identifies were not provided.

In the *P. axillaris* genome, 5 *SUT* and 36 *SWEET* homologous genes are identified (Figure 1 and Figure 2; Table 1)**.** The tissue-specific expression pattern reveals that the flower expresses relatively high levels of members from both *SUT* and *SWEET* families, hinting the important function of sugar transport activity in the reproductive organ. Whereas the 5 *SUT* are expressed at the flower opening stage, *PaSUT1* and *PaSUT2* are notable for the most abundant mRNA at this stage. Evidence obtained by reverse genetic experiments from cucumber and tomato suggests that the orthologous of *SUT1* and *SUT2* are required for sugar availability to support pollen development and pollen tube growth, the loss-of function mutants of which are defeated in fertility [64,65]. On the other hand, although more *SWEET* genes are expressed abundantly in flower opening stage, variation in the expression patterning does exist, for example, *PaSWEET10a* in stage 1 and 2, *PaSWEET6* and *PaSWEET10b* in stage 3. However, their specific roles and regulation are not known yet. Moreover, *PaSWEET13c* represents a constitutively active member during flower development and is the most expressed among the *SWEET* genes in root and reproductive tissues (Figure 2). At the flower opening stage, *PaSWEET13c* is ~10- and ~1.8-fold more abundant in transcript level than the nectar-specific *PaSWEET9c* and second high-abundance *PaSWEET9a*, respectively (Figure 4). Interestingly, *PaSWEET9a* is predicted as a tonoplast transporter based on similarity search (Table S1). The next step experiment would be to generate knock-out mutants of the individual transporter gene for further functional determination.

## 4. Conclusions

In summary, in the model ornamental plant *P. axillaris*, five *SUT/SUC* and thirty-six *SWEET* coding genes are identified in the genome which exhibit divergent expression profiles in tissue-specific and flower developmental stage-associated manners. Active transcriptional activities of several transporter genes are found in the developing flower, more or less correlating with the dynamic changes in soluble sugar contents and the main plant hormone variation. These results provide a starting information for further deciphering sugar-hormone interaction in the context of regulatory mechanism in *Petunia* flower development.

## 5. Materials and Methods

### 5.1. Plant Materials, Growth Conditions and Sample Collections

Seeds of *P. axillaris* (kindly provided by Dr. Hajirezaeiwere, Leibniz Institute of Plant Genetics and Crop Plant Research, Gatersleben, Germany) were surface-sterilized and sown on growing media (vermiculite) in a growth chamber under a photoperiod of 14/10 h of light/dark and 25 °C/22 °C temperature and 65% humidity. After the 7th week of seed germination, the young shoot cuttings were used for clone propagation in new pots. Cuttings were treated with rooting hormone NAA for successful root induction. The plants were further maintained in the chamber.

Samples were collected from various organs and tissues in adult plants propagated from cuttings for total RNA isolation. Pooled samples from 5 individual plants were quickly frozen in liquid nitrogen and stored at −80 °C until use. This sample collection was repeated three times for biological replications. For analysis of flower development, labeled floral buds were followed and sampled on a daily based until corolla was fully open. The collected samples were divided into five defined developmental stages, as specific in the Result Section, in a one-day interval during the flower development until the first day of corolla opening. Flowers from at least 5 plants were pooled and stored as above in −80 °C. Three sets of sampling were used as biological replications. Samples of these five stages of development were used for the analysis of phytohormone and sugar contents, as well as determination of gene expression via quantitative real-time PCR, respectively.

### 5.2. Gene Identification, Sequence Analysis, Phylogenetics and Protein Subcellular Prediction

*Petunia* sucrose transporters *SUT* and *SWEET* homologs were identified by performing a BLAST analysis with the *P. axillaris* v1.6.2 CDS database at Sol Genomics Network [79] using the *A. thaliana* and *Solanum lycopersicum* SUT amino acid sequences, and *A. thaliana* SWEET amino acid sequences as the query sequences, respectively. *A. thaliana* and *S. lycopersicum* SUT, and *A. thaliana* SWEET amino acid sequences were retrieved from the Phytozome database [80].

The exon/intron structure visualizations were generated using The Gene Structure Display Server (GSDS ver. 2.0) [81]. Motifs were identified using the MEME program [82] with the parameters: the maximum number of motifs -10 and optimum width of motifs was set from 6 to 50. Chromosomal locations were obtained using the Mapgene2chrom tool [83].

For phylogenetic analysis, multiple sequence alignments of the SUT and SWEET proteins of *A. thaliana, S. lycopersicum* and *P. axillaris* were performed using the default parameters of MUSCLE [84]. Dendrograms were generated by the MEGA X program using the neighbor-joining (NJ) method with a bootstrap number of 1000 replications. The molecular weight (MW) and isoelectric points (pI) of the presumed SUT and SWEET proteins of *P. axillaris* were predicted by the online ExPASy proteomics server [85].

For protein subcellular prediction, we used three different web-based programs, namely CELLO2GO [86], BUSCA [87] and WoLF PSORT [88] with their default parameter settings. The prediction results are provided in Table S1.

### 5.3. RNA Extraction and Quantitative Real-Time-PCR Analysis

For flower bud tissues, Total RNA was obtained using LabServe Universal RNA kit (Thermo-Fisher Scientific, Shanghai, China) with KingFisher magnetic manipulator (Thermo-Fisher Scientific) following the manufacturer’s instruction. For other plant tissues, RNA was extracted using TransZol UP (Transgen Biotech Co., Ltd., Beijing, China) following the manufacturer’s instruction. Briefly, 300 mg of liquid-nitrogen-ground fine powder was extracted in 800 µL TransZol UP. After adding 160 µL of chloroform, the aqueous phase was separated and precipitation of the RNA was done by mixing with 800 µL isopropanol and pellet was washed with 400 µL of 75% ethanol (prepared with DEPC treated water). After two washes, RNA pellet was air dried and dissolved in 30 µL of RNase-free water. One microgram of total RNA was used in one reaction of cDNA synthesis using TransScript^®^ One-Step gDNA Removal and cDNA Synthesis SuperMix (Transgen Biotech Co., Ltd.) per protocol of the provider.

Quantitative real-time PCR was performed with SYBR Green Master (ROX) (Newbio Industry, China) in a total reaction volume of 15 µl. The PCR program in a Roche Light Cycler96 were set as: 95 °C for 3 min, 40 cycles of amplification (95 °C for 10 s, 60 or 58 °C for 30 s and 72 °C for 20 s) and a final extension at 65 °C for 1 min. Each reaction for every gene was conducted as three-technical replications and we repeated with 3 determinations of independently collected biological samples. Specific PCR primers for *PaSUTs* and *PaSWEETs* genes were designed using online Primer3Plus software [89]. The specificity of the primers was verified using RT-PCR to ensure a single band product and melting-curve detection confirming unique peak. Prior to quantification, annealing temperature optimization was conducted by testing Ta temperatures ranging from 55 to 70 °C. The *PaACTIN2* was used as an endogenous reference gene for the calculation of the 2^(targetCt-referenceCt)^. The primers used are listed in Table S3.

### 5.4. Quantification of Phytohormones Using 6410 Triple Quad LCMS

To quantify phytohormones, floral tissues of five distinct flower developmental stages of *P. axillaris* were thoroughly ground to a fine powder in liquid nitrogen with a cooled mortar and pestle, transferred to the sterilized plastic tubes wrapped in aluminum foil, and immediately kept in liquid nitrogen all the time during grinding of other samples and then stored at −80 °C. For the extraction of phytohormones, 100 mg ground powder was extracted with 900 µL methanol/water (80%, *v*/*v*), to which 100 µL internal standard (i-IAA, i-ABA, i-SA, i-GA1, i-GA4, 10 ng/mL) was added, ultrasonic half-hour at 4 °C, standing for overnight in −20 °C. Afterward, the sample was kept on ice, treated ultrasonically for half-hour at 4 °C, centrifuged at 4 °C and 14,000 rpm for 10 min. The supernatant was collected in a new tube and saved. The precipitate was further extracted by adding 500 µL methanol (80%, *v*/*v*), again with ultrasonic treatment for half-hour at 4 °C. This second extracted supernatant was combined to previously extracted supernatant. The pooled supernatant was reduced in a SpeedVac (Thermo Fisher) to a volume of ~300 µL. To this tube, 700 µL 1% formic acid (*v*/*v*) was added and applied vortex for 1 min, let standing for about 3 h in −20 °C to settle full extraction. For Solid-phase extraction using SPE (Oasis MCX extraction cartridge, 60mg 3mL), sample was activated in 2 mL 70% methanol, 2 mL 0.1M HCl and 2 mL 1% formic acid and then loaded into the column. Afterward, 2 mL of 1% formic acid was added for flushing interference. Acidic and basic fractions were collected separately. For acidic hormones, including IAA, ABA, JA, SA, GA, the elution target fraction was prepared with 2 mL of 70% methanol, while for basic hormone, Tz, the elution target fraction was prepared with 2 mL 5% ammonium hydroxide. After concentrating these fractions to dryness in a SpeedVac (Thermo Fisher), 200 µL methanol (70%, *v*/*v*) was added into both fractions, mixed by vortex, loaded into the glass tubes. Finally, acidic (IAA, ABA, JA, SA, GA1, and GA4) and basic hormones (Zeatin) in these fractions were analyzed by 6410 Triple Quad LCMS.

### 5.5. Quantification of Sugars Using Gas Chromatography—Quadruple Time of Flight Mass Spectrometry (GC-Q ToF MS)

Either fresh or liquid nitrogen-frozen samples were ground to a fine powder with a cooled mortar and pestle in liquid nitrogen. After vacuum drying at −85 °C for 48 h, the powders were stored at −80 °C. For determination of soluble sugars, an amount of 30 mg powdered (dried) sample was taken in a 2 mL centrifugal tube containing 500 μL 75% MeOH solvent (methanol/water, 3:1) and mixed by vortex slightly. Twenty microliters of vanillic acid (5 mg/mL) were added as an internal standard in each tube. The mixture was ultrasound for 30 min on ice and then centrifuged at 14,000 rpm for 10 min at room temperature. The maximum supernatant was then transferred to 1.5 mL tubes and then centrifuged again at 14,000 rpm for 10 min at room temperature. An amount of 150 μL supernatant was then transferred to 2 mL sample vial and placed in a SpeedVac (Thermo Fisher) for vacuum rotary evaporation for 4 h. 80 μL Methoxypyridine solution (42 mg methoxyamine hydrochloride in 2.1 mL pyrimidine, 20 mg/mL) was then added to the dried samples for derivatization and gasification and placed in oven at 80 °C for 20 min. Afterward, the vials were taken out from the oven and 80 μL BSTFA (N,O-Bis{trimethylsilyl}trifluoroacetamide) +1% TMCS (Trimethylchlorosilane) mixture was then added in derived solution of each sample and then placed in oven at 70 °C for 1 h. Extract (~0.2 mL) was then micro filtered and then subjected to nontargeted metabolites analysis with a LECO PegasusHT GC-Q ToF MS, equipped with a capillary tube (Type code: DB-5 MS, Size: 30 m × 0.25 mm × 0.25 μm). The running temperatures for injection was set at 280 °C, capillary temp at 275 °C. The split ratio and flow rate were 10:1 and 1.5 mL/min, respectively.

### 5.6. Statistical Analysis

Statistically significant difference was analyzed using a standard one-way ANVOA followed with a pair-wide Turkey *t*-test under 95% confidence interval, as implemented in Prism GraphPad ver. 8.3.

## Figures and Tables

**Table 1:** Identity of 5 *PaSUTs* and 36 *PaSWEETs* in *P. axillaris* genome.

Gene Mane		Locus ID	Gene Location	Gene Length (bp)	CDS Length (bp)	Peptide Residues	pI
**SUT/SUC Family**							
**1**	*PaSUT1*	Peaxi162Scf00495g00036.1	354,487–359,931	5444	1548	516	5.0
**2**	*PaSUT2*	Peaxi162Scf00503g00419.1	445,731–452,274	6543	1680	560	5.0
**3**	*PaSUT3*	Peaxi162Scf00481g00028.1	281,749–287,120	5371	1521	507	5.0
**4**	*PaSUT4*	Peaxi162Scf00626g00416.1	429,871–441,778	11907	1524	508	5.0
**5**	*PaSUT5*	Peaxi162Scf00268g00137.1	1,330,847–1,335,013	4166	1575	525	5.0
**SWEET Family**							
**1**	*PaSWEET1a*	Peaxi162Scf00263g00161.1	1,672,743–1,674,573	1830	696	232	5.2
**2**	*PaSWEET1b*	Peaxi162Scf00033g00610.1	626,372–627,611	1239	699	233	5.2
**3**	*PaSWEET1c*	Peaxi162Scf00051g00211.1	208,784–211,388	2604	774	258	5.1
**4**	*PaSWEET1d*	Peaxi162Scf00263g00156.1	1,501,206–1,504,332	3126	927	309	5.1
**5**	*PaSWEET1e*	Peaxi162Scf00263g01423.1	1,475,112–1,477,300	2188	891	297	5.1
**6**	*PaSWEET2a*	Peaxi162Scf00111g01241.1	1,263,409–1,265,523	2114	1194	398	5.1
**7**	*PaSWEET2b*	Peaxi162Scf00899g00310.1	296,611–329,600	32,989	705	235	5.2
**8**	*PaSWEET2c*	Peaxi162Scf01337g00018.1	11,448–14,668	3220	705	235	5.2
**9**	*PaSWEET3*	Peaxi162Scf00904g00131.1	169,563–171,965	2402	711	237	5.2
**10**	PaSWEET4 *	Peaxi162Scf00102g01855.1	1,853,060–1,856,259	2021	750	249	5.2
**11**	*PaSWEET5a*	Peaxi162Scf00689g00339.1	307,462–314,776	7314	1167	389	5.0
**12**	*PaSWEET5b*	Peaxi162Scf00017g02829.1	2,794,729–2,795,924	1195	714	238	5.1
**13**	*PaSWEET6*	Peaxi162Scf00129g01027.1	1,045,179–1,046,437	1258	651	217	5.2
**14**	*PaSWEET7*	Peaxi162Scf01051g00019.1	196,117–196,821	704	705	235	5.1
**15**	*PaSWEET8*	Peaxi162Scf00074g00434.1	462,439–465,223	2784	612	204	5.2
**16**	*PaSWEET9a*	Peaxi162Scf00386g00924.1	926,235–928,356	2121	792	264	5.2
**17**	*PaSWEET9b*	Peaxi162Scf00028g00063.1	675,430–685,040	9610	685	228	5.2
**18**	*PaSWEET9c(NEC1)*	Peaxi162Scf00303g00004.1	55,308–57,418	2110	843	281	5.2
**19**	*PaSWEET10a*	Peaxi162Scf01039g00027.1	73,727–75,238	1511	852	284	5.1
**20**	*PaSWEET10b*	Peaxi162Scf00067g00107.1	995,051–996,843	1792	816	272	5.1
**21**	PaSWEET11a *	Peaxi162Scf00516g00125.1_126	165,635–167,699	1948	879	292	5.2
**22**	*PaSWEET11b*	Peaxi162Scf00909g00223.1	308,317–310,150	1833	825	275	5.1
**23**	*PaSWEET12a*	Peaxi162Scf00516g00033.1	55,540–65,536	9996	1599	533	5.0
**24**	*PaSWEET12b*	Peaxi162Scf01064g00253.1	289,074–307,657	18,583	2304	768	4.9
**25**	*PaSWEET13a*	Peaxi162Scf00502g00058.1	562,962–564,843	1881	750	250	5.2
**26**	*PaSWEET13b*	Peaxi162Scf00358g01310.1	1,321,846–1,324,432	2586	867	289	5.1
**27**	*PaSWEET13c*	Peaxi162Scf00516g00122.1	104,493–106,655	2162	852	284	5.1
**28**	*PaSWEET14a*	Peaxi162Scf00516g00124.1	133,860–155,056	21,196	2355	785	4.9
**29**	*PaSWEET14b*	Peaxi162Scf00647g00518.1	595,043–596,818	1775	984	328	5.1
**30**	*PaSWEET14c*	Peaxi162Scf00516g00025.1	78,107–79,474	1367	717	239	5.2
**31**	*PaSWEET14d*	Peaxi162Scf00516g00012.1	119,256–121,066	1810	690	230	5.2
**32**	*PaSWEET15a*	Peaxi162Scf00128g01632.1	1,656,934–1,660,444	3510	966	322	5.1
**33**	*PaSWEET15b*	Peaxi162Scf00255g00006.1	30,836–33,121	2285	816	272	5.1
**34**	*PaSWEET16a*	Peaxi162Scf00471g01110.1	1,106,818–1,108,891	2073	618	206	5.2
**35**	*PaSWEET16b*	Peaxi162Scf00037g01416.1	1,392,651–1,396,158	3507	723	241	5.2
**36**	*PaSWEET17*	Peaxi162Scf00037g01417.1	1,401,046–1,404,180	3134	894	298	5.1

* mRNA sequences of these two genes have been corrected via prediction with FGENESH [60], the corrected mRNA sequences differ from the current version of genome annotations.

## Supplementary Materials

The following are available online at https://www.mdpi.com/2223-7747/9/12/1770/s1, Figure S1: Genome location of the 5 *PaSUT* and the 36 *PaSWEET* genes in P. axillaris, Figure S2: The intron- exon structure of the *PaSUT* and *PaSWEET* genes, Figure S3: Transverse sectioning of the *Petunia axillaris* flowers in the five stages, Table S1: Prediction of subcellular localization of the sugar transporters, Table S2: Statistics of *PaSWEET* gene expression during flower development quantified via qRT-PCR, Table S3: List of primers used for qRT-PCR.
